# Membrane-Driven Dimerization of the Peripheral Membrane Protein KRAS: Implications for Downstream Signaling

**DOI:** 10.3390/ijms25052530

**Published:** 2024-02-21

**Authors:** Ki-Young Lee

**Affiliations:** Department of Pharmacy, College of Pharmacy and Institute of Pharmaceutical Sciences, CHA University, Pocheon-si 11160, Gyeonggi-Do, Republic of Korea; kyleejh@cha.ac.kr

**Keywords:** KRAS, peripheral membrane protein, dimerization, nanodisc, paramagnetic relaxation enhancement (PRE)

## Abstract

Transient homo-dimerization of the RAS GTPase at the plasma membrane has been shown to promote the mitogen-activated protein kinase (MAPK) signaling pathway essential for cell proliferation and oncogenesis. To date, numerous crystallographic studies have focused on the well-defined GTPase domains of RAS isoforms, which lack the disordered C-terminal membrane anchor, thus providing limited structural insight into membrane-bound RAS molecules. Recently, lipid-bilayer nanodisc platforms and paramagnetic relaxation enhancement (PRE) analyses have revealed several distinct structures of the membrane-anchored homodimers of KRAS, an isoform that is most frequently mutated in human cancers. The KRAS dimerization interface is highly plastic and altered by biologically relevant conditions, including oncogenic mutations, the nucleotide states of the protein, and the lipid composition. Notably, PRE-derived structures of KRAS homodimers on the membrane substantially differ in terms of the relative orientation of the protomers at an “α–α” dimer interface comprising two α4–α5 regions. This interface plasticity along with the altered orientations of KRAS on the membrane impact the accessibility of KRAS to downstream effectors and regulatory proteins. Further, nanodisc platforms used to drive KRAS dimerization can be used to screen potential anticancer drugs that target membrane-bound RAS dimers and probe their structural mechanism of action.

## 1. Introduction

Human membrane proteins comprise approximately one third of the proteome of cells and one half of the current drug targets [1,2,3]. A variety of protein–protein interactions in or on the surface of membranes underlie many fundamental biological processes, including signal transduction, sensing, bioenergetics, and organization [4]. However, despite the biological and therapeutic importance of membrane multiprotein complexes, it has been challenging to determine their high-resolution structures, especially for the low-affinity self-assembly of signaling-competent membrane proteins. These complexes are often spatiotemporally formed in response to extracellular stimuli or intracellular cues and promote the activation of their downstream effectors, which is a well-known molecular process to initiate intracellular signaling pathways responsible for cell proliferation, differentiation, or apoptosis [5,6,7]. 

**Figure 1:**
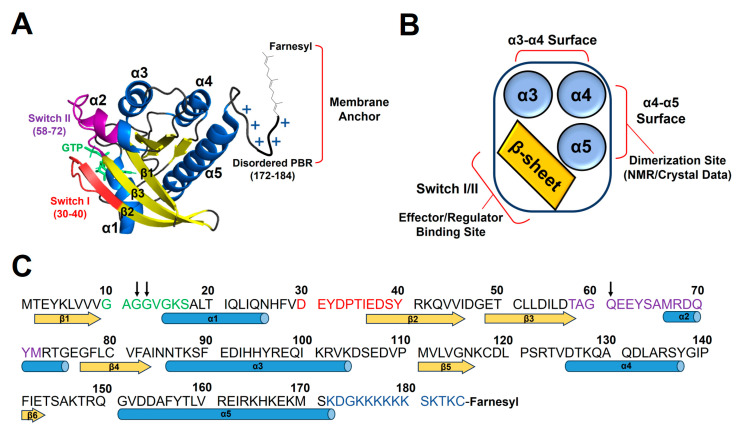
The structure and functional sites of KRAS. (**A**) Crystal structure of KRAS in the active GTPγS (a non-hydrolyzable GTP analog)-bound state (PDB ID: 4DSO). (**B**) Schematic of functional sites of KRAS. KRAS dimerization involves several different ‘α4–α5’ surfaces, as determined using NMR and X-ray crystallography, and the β-sheet region including Switch I and II sites binds to RAS effectors (e.g., RAF, PI3Kγ) or regulators (e.g., GAP, GEF). (**C**) The amino acid sequence of KRAS. Residues 12, 13, and 61, whose single-point mutations are associated with many human cancers, are indicated as black arrows. Secondary structure content is indicated at the bottom of the sequence with α-helices (blue) and β-strands (yellow). Nucleotide binds to a conserved phosphate-binding loop (P-loop comprising residues 10–17, green). Switch I (residues 30–40) and Switch II (residues 58–72) regions and disordered C-terminal polybasic region (PBR, residues 172–184) are colored red, violet, and blue, respectively.

Among many peripheral membrane proteins, the RAS (rat sarcoma viral oncogene homolog) isoforms, KRAS, HRAS, and NRAS, downstream of receptor tyrosine kinases act as central hubs to relay extracellular ligand-stimulated signals to cytoplasmic multiple signaling pathways for cell proliferation and differentiation [8,9]. RAS proteins have the highly homologous GTPase domain (residues 1–165) that cycles between the active GTP- and inactive GDP-bound states, with the disordered C-terminal hypervariable region (residues 166–185). The GTPase domain is composed of a six-stranded central β-sheet surrounded by five α-helices (Figure 1). KRAS has the polybasic region (PBR, residues 172–184) enriched in lysines, which can associate favorably with membranes containing anionic lipids, phosphatidylserine (PS), phosphatidylinositol phosphate (PIP), or phosphatidic acid (PA) [10,11]. Additionally, GTP binding induces a conformational change in Switch I and II regions near the β-sheet effector/regulator binding site of RAS and facilitates the scaffolding and activation of downstream effectors [12,13]. The α4–α5 region on the opposite side of the β-sheet region is involved in dimerization or crystallographic contact of KRAS (Figure 1) [14,15,16]. Notably, single-point mutations occur near the nucleotide-binding pocket of RAS, which impairs the GTPase activity and locks the protein in a constitutively GTP-bound state. These gain-of-function mutations are known as some of the most prevalent oncogenic drivers, occurring in a quarter of human cancers [17]. Moreover, KRAS is mutated at position 12 (G12X) in ~80% of RAS-driven cancers [18]. 

A role for the homo-dimerization of RAS on the membrane surface in the activation of downstream RAF (rapidly accelerated fibrosarcoma) kinases was proposed two decades ago [19], and increasing evidence has suggested that transient RAS dimers at the plasma membrane could serve as the basic units to recruit and activate downstream effectors (e.g., RAF kinases) [16,20,21,22,23,24]. Although dimers are the predominant oligomeric form at the physiological expression level [20], it has been demonstrated that dimerization is a prerequisite process for the formation of higher-order oligomers (i.e., nanoclusters) comprising approximately four to six RAS monomers [25]. Advances in fluorescence-based approaches and electron microscopy have provided insights into RAS nanoclustering in cells, yet obtaining the residue-specific structural information about nanoclusters remains challenging [26,27,28,29]. It should be noted that these methods have been carried out using nonnative KRAS (~21 kDa) samples tagged with relatively large fluorescent proteins (~27 kDa), which may affect the orientation of KRAS with respect to the membrane and KRAS self-association. In addition, most of the crystal structures of RAS have been determined regarding the highly conserved GTPase domains of the RAS isoforms. Notably, these GTPase domains lack the disordered C-terminal hypervariable region (HVR) responsible for membrane association, which provides limited structural information on membrane-associated RAS dimers. Several molecular dynamics (MD) studies have proposed a diversity of RAS dimer structures with the α3–α4, α4–α5, and β-sheet regions [30,31,32,33,34], likely due to different convergence criteria in MD simulations (several hundred nanosecond to low microsecond range) and/or to challenges in incorporating the dynamic nature of the RAS interfaces into the simulation processes. Consequently, this lack of consistent structural models has warranted rigorous experimental set-ups for the structure determination of membrane-bound RAS dimers using high-resolution biophysical tools and lipid nanoparticles that provide native-like membrane environments.

**Figure 2:**
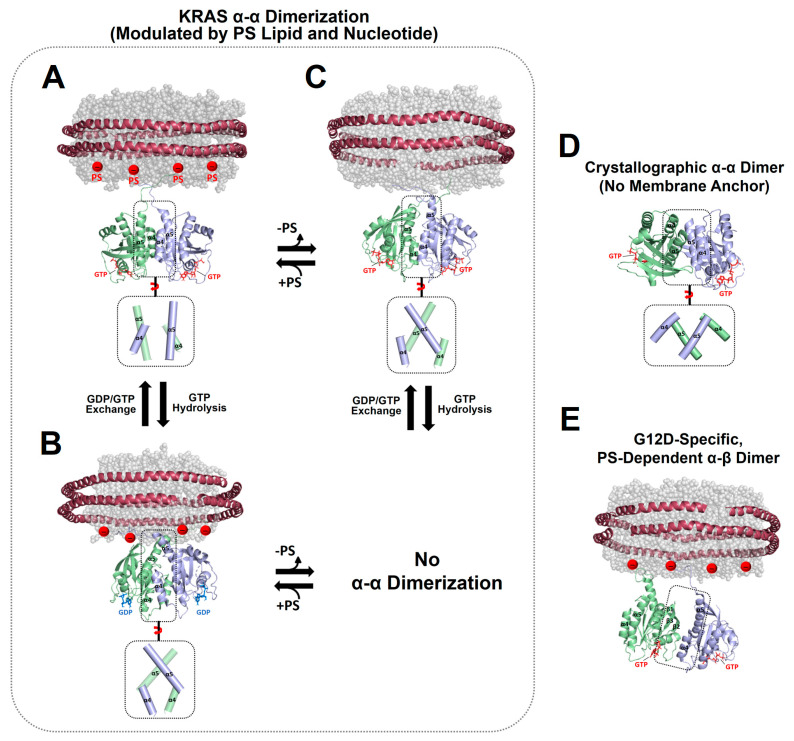
Representative NMR-driven structures of symmetric α–α dimers of full-length KRAS in the GTP-bound (**A**) and GDP-bound (**B**) states on anionic membranes containing phosphatidylserine (PS) lipids (PDB IDs: 6W4E and 6W4F) and the α–α dimer of GTP-bound KRAS on a neutral membrane lacking PS (**C**). The dimerization of GDP-bound KRAS is unfavorable on the neutral membrane. (**D**) Crystal structure of a symmetric α–α dimer of membrane-free KRAS lacking the disordered C-terminal hypervariable region (HVR) responsible for membrane association (PDB ID: 5VQ2). (**E**) The representative NMR-driven structure of an asymmetric α–β dimer of the full-length KRAS-G12D mutant on an anionic membrane containing PS (PDB ID: 7RSE). This mutant also forms the symmetric α–α dimer shared with wild-type KRAS. Differences in the relative orientation of the protomers at the α–α dimer interfaces are highlighted in dotted boxes.

**Figure 3:**
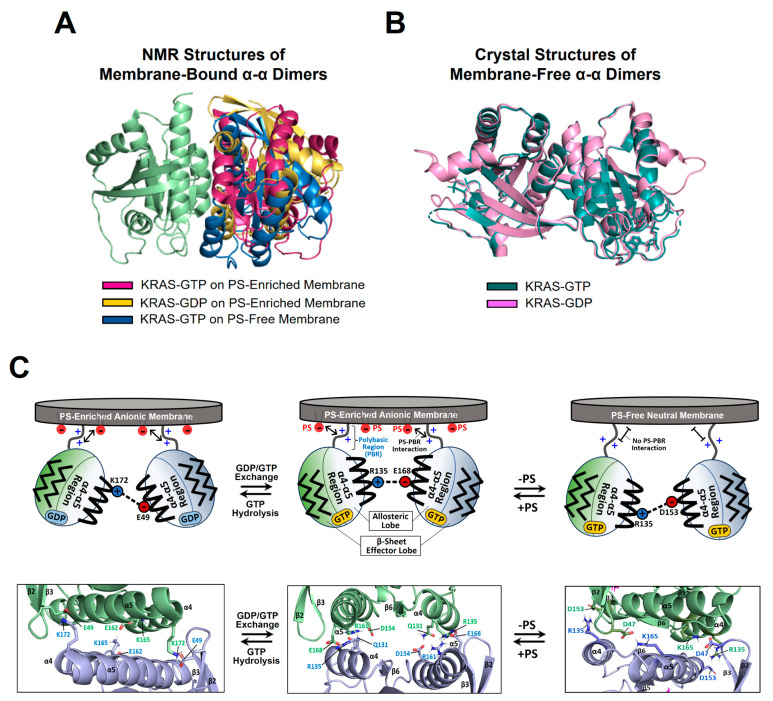
Plasticity of the KRAS dimer interface. (**A**) Overlay between representative NMR-driven structures of full-length KRAS-GTP dimers on both anionic (phosphatidylserine [PS]-enriched) and neutral (PS-free) membranes and the full-length KRAS-GDP dimer on the anionic membrane (PDB IDs: 6W4E and 6W4F). For clarity, only the GTPase domains (residues 1–172) of the structures are shown. One protomer in each KRAS dimer is overlaid to clearly show the different arrangements of the opposing protomers. (**B**) Overlay between the crystal structures of membrane-free KRAS dimers in both GTP- and GDP-bound states lacking the disordered C-terminal hypervariable region (HVR) (PDB IDs: 5VQ2 and 5W22). (**C**) Promiscuous intermolecular interactions at the α–α dimer interface of full-length KRAS on the membrane. The KRAS dimer interfaces involve intermolecular electrostatic interactions that are modulated by the nucleotide-bound state of KRAS and the lipid composition of the membrane. Representative electrostatic interactions with a cut-off distance < 3.5 Å at the dimer interface, validated by interface-specific mutagenesis, are schematically indicated.

**Figure 4:**
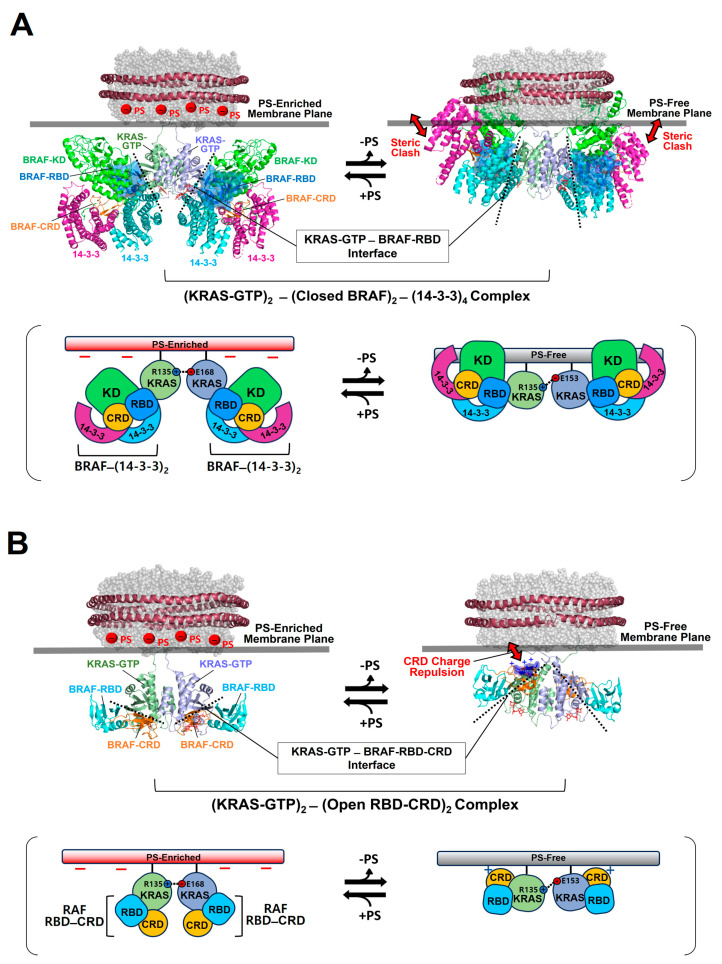
Overlay of representative NMR-derived structures of KRAS-GTP dimers on membranes containing or lacking phosphatidylserine (PS) with the structures of KRAS in complex with the autoinhibited BRAF–14-3-3 complex (**A**) and the RAS binding domain (RBD)–cysteine rich domain (CRD) of active BRAF (**B**). The arrow represents steric clash or charge repulsion between the effector and the membrane surface. The complex models are reconstituted based on the cryo-electron microscopy (EM) structure of the BRAF–14-3-3 complex (PDB ID: 7MFE), the crystal structure of KRAS in complex with the RBD–CRD (PDB ID: 6XI7), and the NMR-derived structure of the KRAS-GTP dimer on the membrane (PDB ID: 6W4E).

**Figure 5:**
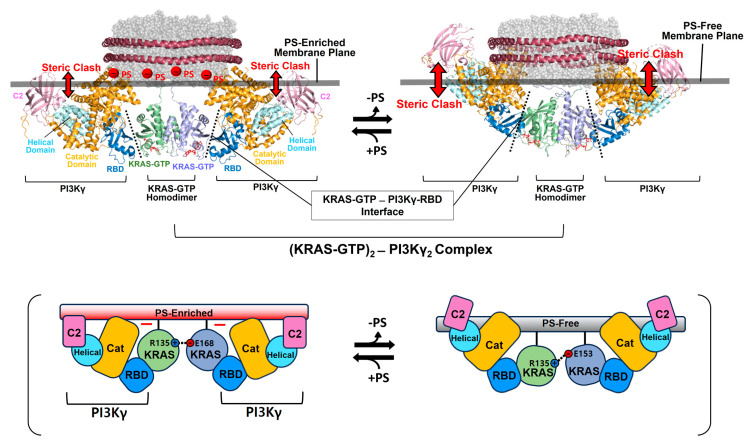
Overlay of representative NMR-derived structures of KRAS-GTP dimers on membranes containing or lacking phosphatidylserine (PS) with the structures of KRAS in complex with phosphoinositide 3-kinase gamma (PI3Kγ). The arrow represents steric clash between the effector and the membrane surface. The complex models are reconstituted based on the crystal structures of KRAS in complex with PI3Kγ (PDB ID: 1HE8) and the NMR-derived structure of the KRAS-GTP dimer on the membrane (PDB ID: 6W4E).

**Figure 6:**
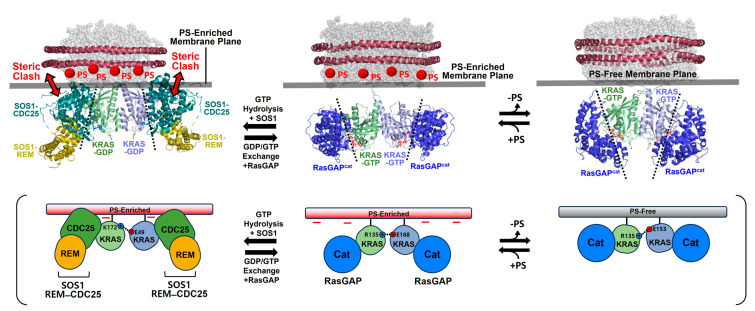
Structural models of membrane-bound KRAS dimers in complex with the catalytic domains of son of sevenless homolog 1 (SOS1) and Ras GTPase activating protein (RasGAP). Representative NMR-derived structures of KRAS-GTP dimers on membranes containing or lacking phosphatidylserine (PS) are overlaid with the crystal structure of KRAS in complex with the catalytic domain of RasGAP (PDB ID: 1WQ1). The representative NMR-derived structure of the KRAS-GDP dimer on an anionic membrane containing PS is overlaid with the crystal structure of KRAS in complex with the REM–CDC25 domain of SOS1 (PDB ID: 6EPL).

**Figure 7:**
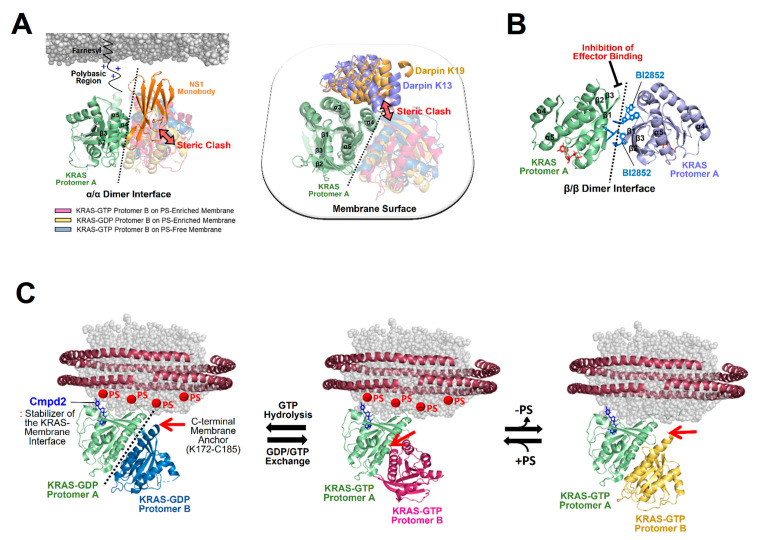
The structural mechanism of KRAS dimerization modulators. (**A**) Overlay of representative NMR-driven structures of KRAS α–α dimers with the crystal structures of KRAS in complex with the following synthetic proteins: the NS1 monobody (PDB ID: 5E95) and designed ankyrin repeat proteins (DARPins) K13 (PDB ID: 6H46) and K19 (PDB ID: 6H47). These KRAS binders cause steric clashes with KRAS protomers in dimers, as indicated by the arrows, and act as competitive inhibitors of KRAS dimerization. One protomer in each KRAS dimer is overlaid to clearly show the different arrangements of the opposing protomers. (**B**) BI2852-mediated formation of an inactive β-β dimer of KRAS-G12D (PDB ID: 6GJ8) in which the effector binding sites at the β-interfaces are inaccessible to RAS effectors. (**C**) Overlay of the representative NMR-driven structure of membrane-anchored KRAS in complex with the protein–membrane interaction (PMI) stabilizer Cmpd2 (PDB ID: 6CC9) with representative NMR-driven structures of KRAS-GTP α–α dimers on phosphatidylserine (PS)-enriched anionic or PS-free neutral membranes and the KRAS-GDP α–α dimer on a PS-enriched anionic membrane. Red arrows represent the positions to which the disordered C-terminal membrane anchors (K172-C185) of KRAS are attached.

Several types of lipid-based nanoparticles with different shapes and sizes, such as micelles, bicelles, liposomes, and nanodiscs, have been extensively used to elucidate the structural basis and biochemical properties of membrane-bound proteins in the structural biology field [35,36]. Among these nanoparticles, discoidal nanodiscs have increasingly gained attention because nanodiscs provide native lipid-bilayer membranes and enable the preparation of highly stable, homogeneous protein–lipid samples suitable for structural analyses using cryo-electron microscopy (EM) and nuclear magnetic resonance (NMR) [37,38,39,40]. The lipid bilayer is encircled by two copies of membrane scaffold protein (MSP), of which variants can define the size of the nanodisc lipid surface. Recently, nanodisc-bound KRAS molecules have been subjected to paramagnetic relaxation enhancement (PRE)–NMR experiments, revealing the distinct structures of KRAS dimers on the membrane along with residue-specific information on the KRAS dimerization interface and the KRAS-membrane interface [14,15,41]. The 20 energy-minimized NMR conformers for each dimer are well converged with an RMSD of ~1 Å (the lowest energy conformers are used as representatives in Figure 2, Figure 3, Figure 4, Figure 5, Figure 6 and Figure 7). The lipid surface diameter of the MSP1D1-type nanodisc (~76 Å) used was sufficient to accommodate two KRAS molecules (~36 Å per monomer) [42,43]. KRAS molecules anchored to a nanodisc complex with a high molecular weight of >100 kDa have exhibited good reference NMR spectra without severe line broadening [14,41], suggesting that membrane-anchored KRAS is highly dynamic on the high picosecond to nanosecond time scale. PRE experiments have been developed as highly sensitive tools for the visualization of sparsely populated, low affinity protein complexes, as the PRE effect is strong and highly dependent on the intermolecular distance between the paramagnetic center and observed nucleus [44,45]. Technically, paramagnetic spin labels including unpaired electrons (e.g., radical ions) are introduced to a sample, which causes transversal PRE (Γ_2_) and resonance broadening of the NMR probes in proximity of the paramagnetic spin. Specifically, Γ_2_ is calculated as the difference between the two transverse relaxation rates (R_2_) for the paramagnetic and diamagnetic samples (Γ_2_ = R_2,para_ − R_2,dia_). The degrees of Γ_2_ can be converted to long-range distance restraints (up to ~24 Å) at the protein–protein or protein–ligand interfaces for structure calculation. In the case of transient complex formation in fast exchange on the PRE time scale, the apparent Γ_2_ could represent the fraction of an isotopically labeled protein that is bound to a spin-labeled protein. 

Here, we emphasize the dynamic self-association between a pair of KRAS protomers on the membrane. Intermolecular interactions within the KRAS dimer interface are highly plastic and reversible and altered by the nucleotide and mutation states of KRAS and the lipid composition of the membrane. This condition-modulated KRAS dimerization will be discussed in this review, providing structural insights into the KRAS-dependent activation of downstream effectors. Such dynamic interactions are applicable to many other peripheral membrane proteins, including small GTPases, that form transient dimers or higher-order oligomers as functional units in response to variable membrane environments in cells. 

## 2. Promiscuous Dimerization Interfaces of KRAS on the Membrane

Accumulating data have demonstrated the presence of RAS dimers and less favorable, larger oligomers on the membrane [10,16,20,21,22,23,24,25,46]. Recently, nanodisc-based systems have been developed to determine the atomic-resolution structures of KRAS homodimers in both active and inactive states on membranes containing or lacking anionic PS lipids [14,15,41]. These structures exhibit three different types of symmetric “α–α” dimer interfaces that involve two α4–α5 surfaces (α-interface) of the protomers and an asymmetric “α–β” dimer interface between the α-interface and the β-sheet region (β-interface) (Figure 2). The KRAS G12D mutant, one of the most prevalent oncogenic mutants, can form an asymmetric α–β dimer in addition to the α–α interface shared with wild-type KRAS-GTP [41]. However, PS depletion abrogates the additional α–β interaction of the G12D mutant, consistent with previous data indicating that PS lipids promote the formation of KRAS dimers and higher-order oligomers on the plasma membrane in cells [10,11]. The α–α dimers of KRAS on the membrane substantially differ in terms of the relative orientation of the protomers at the dimer interface depending on the nucleotide-bound state of KRAS and whether PS lipids are incorporated into the membrane (Figure 2 and Figure 3). These interfacial orientations are distinct from those of the crystallographic α–α dimers of the membrane-free RAS in both the GTP- and GDP-loaded states, which have essentially identical dimer interfaces (Figure 3B). However, it is unclear whether the crystallographic dimers can be formed in other biological environments or are simply artifacts induced by crystal packing, which is supported by the observation that KRAS exists in a monomeric state in solution [14,41]. 

The structure of the homodimer of KRAS-GTPγS (a non-hydrolyzable GTP analogue, henceforth referred to as GTP) on an anionic membrane containing PS is stabilized mainly by intermolecular electrostatic interactions between (1) Q131 and D154, (2) Q131 and R161, and (3) R135 and E168 (Figure 3C) [14]. The KRAS GTPase domain can undergo GTP hydrolysis to generate the GDP-bound state, which appears to induce translation and rotation of the protomers at the α–α dimer interface. The KRAS-GDP dimer interface involves two key interactions between (1) E49 and K172 and (2) E162 and K165 (Figure 3C) [14]. In the presence of a charge-neutral membrane lacking PS, KRAS-GTP dimerization occurs with a new α–α dimer interface that involves intermolecular electrostatic interactions between (1) R135 and D153 and (2) D47 and K165 (Figure 3C) [15], whereas dimerization of GDP-bound KRAS on the neutral membrane is unfavorable [15]. The additional α–β dimer interface of the KRAS G12D mutant involves electrostatic interactions between (1) R135 at the α interface and D33 or D38 at the β interface and (2) R128 at the α interface and D33 at the β interface [41]. 

Interface-specific mutagenesis experiments have validated the plastic intermolecular interactions at the α–α dimerization interface of KRAS, unveiling that R135 of one KRAS protomer in the GTP-bound state can form alternate salt bridges with D153 and E168 of the opposing protomer in the same state (Figure 3C) [15]. The two intermolecular interactions can co-exist in equilibrium, which is shifted toward the R135-E168 interaction in the presence of an anionic membrane containing PS. Consistent with the structures of the α–α dimers of GTP-bound KRAS, the introduction of single charge-reversal mutations at key points of the KRAS dimerization interface was shown to be sufficient to disrupt the corresponding dimer [14,15]. The R135E mutants do not self-associate regardless of the presence of PS in the membrane, and D153K and E168R selectively disrupt dimers on neutral and anionic membranes, respectively, although these mutations do not impact the formation of dimers on the alternative membrane [15]. Double charge-reversal mutations were introduced into both protomers to selectively restore the charge complementarity at the α–α dimer interfaces to mediate KRAS dimerization [15]. Consequently, the R135E/D153K mutant selectively restored the KRAS dimer on a neutral membrane lacking PS, while the PS-specific dimer conformation was inhibited by this double mutation. This dimerization specificity was reversed by the R135E/R168E double mutant that recovered the electrostatic interaction in the presence of PS. In addition, the presence of a higher salt concentration (i.e., higher ion strength) was found to reduce the formation of KRAS dimers on the membrane and to induce the equilibrium shift toward the R135–D153 interaction, as observed in the absence of PS [15]. Specifically, ion strength may affect intermolecular electrostatic interactions within the KRAS dimer interface and between the anionic head groups of PS lipids in the membrane and the C-terminal PBR of KRAS protomers. These results indicate that the dimerization process of membrane-anchored KRAS is mainly electrostatic. Consistent with the structures of two mutually exclusive dimers of KRAS-G12D, the interface-specific mutations D38K or E168R of KRAS-G12D induce no dimerization mediated by the α–β and α–α interfaces, respectively, while preserving the alternate dimer species [41]. Overall, appreciable effects of interface-specific mutagenesis and ion strength on KRAS dimerization suggest a delicately balanced conformational equilibrium between KRAS dimers involving promiscuous electrostatic interactions at the dimer interfaces.

The formation of multiple α–α dimer interfaces appears to be achieved by translation and rotation of the protomers at the dimer interface. This plasticity may be facilitated by the nature of the α-interface on the “flat” side of the GTPase domain of KRAS and/or by delicately formed intermolecular interactions that are sensitive to changes in the nucleotide and mutation states of KRAS and the lipid composition of the membrane. Notably, the electrostatic interactions within the α–α dimer interface of KRAS are altered by the lipid composition [15], demonstrating allosteric communication between the dimerization specificity of the α-interface and the membrane association of the C-terminal membrane anchor. This allostery involves new functional dynamics of full-length RAS, in addition to the previously well-known conformational change of the switch I and II regions upon the GDP/GTP cycling [12]. RAS proteins have evolved to adopt the conformational flexibility sensitive to the nucleotide binding in response to extracellular signals, and this flexibility may also allow local conformational changes at the KRAS dimerization interface (α4–α5 region) upon the electrostatic interaction between PS lipids and the disordered C-terminal PBR extending from the α5 helix of KRAS. 

KRAS self-associations may be promoted by the reduced dimensionality of KRAS diffusion on the limited surface of nanodiscs, which resemble cellular membrane domains with specific lipid compositions that enhance the effective local concentration of the lipid-bound proteins. In addition, membrane association may induce dimerization-competent orientations of KRAS monomers on the membrane, and dimerization may in turn alter these orientations and the effector accessibility of KRAS. However, it remains to be extensively explored how the KRAS dimerization interface and the KRAS-membrane orientation are influenced by other oncogenic mutations, primarily occurring at Gly12, Gly13, and Gln61, and the post-translational modifications of KRAS and other lipid compositions in the membrane. These studies will provide vital platforms to elucidate the structural basis of KRAS dimerization and signaling in many physiological conditions. 

## 3. Implication of KRAS Membrane Orientations in Downstream Signaling 

Many cellular signaling events take place at the membrane surface, which provides a platform that facilitates the functional assembly of signaling proteins in response to extracellular stimuli or intracellular cues [4,5]. Membranes act as active participants in modulating KRAS dimerization, concomitantly occurring with altered KRAS membrane orientations [14,15,41], which may generate a broad conformational spectrum of KRAS on the membrane. Membrane binding may promote KRAS orientations and/or conformations that enable dimerization and effector binding. Previous PRE experiments using Gd^3+^-associated membranes have demonstrated that KRAS monomers adopt certain preferred orientations on the membrane and have shown how these KRAS orientations are altered by dimerization, depending on the nucleotide and mutation states of KRAS and the PS composition in the membrane [14,15,41]. Some disease-associated mutants of the KRAS monomer were shown to favor an orientation in which the effector binding site at the β-interface is exposed and accessible to the RAS binding domain (RBD) of RAF [47]. In addition, wild-type KRAS monomers, when in complex with the RBD-cysteine rich domain (CRD) of RAF, can adopt the exposed state of the KRAS dimerization site, which facilitates the dimer-mediated activation of downstream RAF effectors [48]. Homo-dimerization of GTP-bound KRAS promotes the effector binding site exposure at the β-interface with respect to the membrane either containing or lacking anionic PS lipids [14,15]. Hence, this orientational change allows productive interactions between KRAS and effectors. 

Recently, cryo-EM structures of the autoinhibited state of BRAF have been deter-mined, showing that a dimer of the 14-3-3 protein (binder of many signaling proteins) bridges the N-terminal and C-terminal phosphorylated sites of BRAF, thus preventing dimerization of the kinase domain (KD) and sequestering the CRD [49]. To disrupt the autoinhibited conformation, the RBD that is attached to the N-terminus of the CRD would bind to KRAS and lead to the exposure of the N-terminal 14-3-3-binding site of BRAF and dephosphorylation at Ser259. This open conformation then promotes the formation of an active KD dimer, which is further stabilized by the 14-3-3 dimer anchoring the C-terminal phosphorylated sites of the BRAF protomers. KD dimerization and BRAF activation would be facilitated by membrane-bound KRAS dimers which bind to the RBDs and foster the recruitment of two adjacent BRAF molecules and the formation of the KRAS–RAF hetero-tetrameric signaling complex. This mechanism has been supported by the observation that RAF activation is confined to RAS nanoclusters likely comprising dimers and higher-order oligomers [10,20,22,50]. An alternate mechanistic model is that this complex is formed by the dimerization of preformed KRAS–RAF heterodimers using allosteric communication by which RBD binding to the β-interface of KRAS promotes the α–α dimerization of KRAS [51]. 

To gain structural insights into the activation of autoinhibited RAF in a closed conformation by membrane-bound KRAS dimers, we constructed the structural models for this autoinhibited state in a complex with KRAS-GTP dimers on the membrane (Figure 4A).

The binding of RAF to KRAS-GTP protomers is compatible with the structure of the KRAS-GTP dimer on an anionic membrane containing PS, whereas the formation of this KRAS–RAF complex clashes with a neutral membrane lacking PS. These structural models suggest that the activation of autoinhibited BRAF by KRAS dimers is promoted in the presence of PS-enriched domains in the membrane. Activated RAFs in an open conformation still bind to two KRAS protomers on the membrane, while the two KDs of RAF are scaffolded by the 14-3-3 dimer and dimerize to function in the cytosol. In the structure of the autoinhibited state of RAF, the RBD is relatively exposed compared to the CRD buried between the KD and the 14-3-3 protein. The RBD–CRD is shown to bind to a region at the β-interface of RAS in the crystal structure of the RAS-RBD–CRD complex [52,53]. These structures suggest that, for the KRAS–RAF interaction, the RBD–CRD would be released from the closed state of RAF by the initial binding of the RBD to KRAS protomers in dimers. The structure of the KRAS-GTP dimer on a PS-enriched, anionic membrane is compatible with the binding of two open RBD–CRDs to the effector binding sites at the β-interfaces on the opposite side of the α–α dimer interface (Figure 4B). In contrast, the formation of this KRAS-RBD–CRD complex on a PS-free, neutral membrane appears to be unfavorable because the basic region of the CRD may cause electrostatic repulsion with the membrane surface, as shown in the structural model (Figure 4B). Notably, it is possible that the CRD affinity for the membrane influences the KRAS dimer orientation along with changes in KRAS-CRD or KRAS–KRAS interactions. Consistent with this possibility, recent MD studies have suggested multiple conformations of the KRAS homodimer in complex with the RBD–CRD on the membrane, with the highly dynamic CRD that can bind to both KRAS and the membrane [54,55]. By contrast, the structures of membrane-bound KRAS dimers are incompatible with the binding of phosphoinositide 3-kinase gamma (PI3Kγ) effectors to KRAS protomers, regardless of the presence of PS in the membrane (Figure 5), consistent with the observation that PI3K activation requires a single RAS interaction and is not promoted by RAS dimerization [56,57]. The dimerization of GDP-bound KRAS moves the effector binding sites closer to the membrane [14]; however, the functional role of this dimerization in RAS signaling remains unknown. 

The propensity to form assemblies involving KRAS–KRAS and KRAS–effector interactions should be altered in the context of protein–protein interaction networks in multiple signaling pathways with RAS as a hub protein. For instance, the assembly process for a signaling-competent complex between KRAS and RAF dimers may be affected by the relative stoichiometry between KRAS, other RAS family proteins, RAFs, other downstream effectors, and upstream regulators (i.e., GEFs and GAPs) as well as by their expression levels that vary in many types and stages of normal or RAS-driven cancer cells [58,59,60,61]. These cells may also generate different membrane environments. Notably, plasma membrane depolarization in excitable cells has been demonstrated to generate PS-clustered membrane domains to promote the KRAS assembly and mitogen-activated protein kinase (MAPK) signaling [62]. KRAS dimerization and the KRAS–RAF interaction on membrane domains of a specific lipid composition (e.g., PS-enriched domains) may synergistically promote a KRAS-RAF-14-3-3 complex that would propagate MAPK signaling. 

## 4. Implication of KRAS Membrane Orientations in GTPase Cycling 

The dimerization-dependent modulation of membrane orientations may also affect the accessibility of KRAS to key regulators involved in GTPase cycling between the GTP- and GDP-bound states of KRAS. The structure of a KRAS-GDP dimer on an anionic membrane containing PS appears incompatible with the binding of the dimer to the catalytic REM–CDC25 domains of the guanine nucleotide exchange factor (GEF) son of sevenless homolog 1 (SOS1) [63] (Figure 6), which accelerates GDP/GTP exchange to produce the active GTP-bound state of KRAS. The SOS1-binding site at the β-interface of KRAS-GDP becomes close to the membrane upon dimerization [14], suggesting that KRAS dimers do not favor the SOS1-mediated nucleotide exchange rate. In contrast, docking of the catalytic domain of the p120 RAS GTPase activating protein (GAP^cat^) [64] is compatible with the structure of KRAS-GTP dimers on membranes either containing or lacking PS (Figure 6).

Dimerization promotes the exposure of the β-interface involved in the activation of GAPs and increases the inactive GDP-bound state of KRAS. GAPs and RAS effectors likely compete to bind to the exposed β-interfaces of KRAS-GTP dimers. The β-interface in the asymmetric α–β dimer, which is specific to KRAS-G12D [41], is not accessible for interaction with RAS effectors and GAPs. This α–β dimerization was shown to be weaker in a nonspecific manner (~1 mM *K*_D_) than the α–α interaction [41], and thus, it is likely that the α–β interaction is readily outcompeted by RAS effectors with much higher affinity (nanomolar *K*_D_) [13]. The transient α–β interaction would promote KRAS molecules in proximity and/or be directly involved in the formation of weakly associated KRAS oligomers on a membrane lipid domain, enhancing the proximity-driven dimerization and activation of downstream RAF kinases [65], but not PI3Ks [56,57].

## 5. Modulators of KRAS Dimerization

The structures of KRAS dimers in complex with RAS effectors and regulators on the membrane are opening new avenues for therapeutic inhibition of the KRAS-mediated effector activation and the regulator-assisted formation of active, GTP-bound KRAS. KRAS is known as one of the most highly mutated proteins in human cancers, and KRAS mutants are well-established drivers of many tumorigenic processes [17]. However, despite decades of intensive drug discovery efforts, KRAS has proven to be an exceptionally challenging drug target [66,67]. The first clinical inhibitor of mutant KRAS, a mutation-specific covalent inhibitor of KRAS G12C, stabilizes the inactive GDP-bound state and has shown promise [68]. However, this covalent mechanism of action is limited because G12C represents only a small fraction of KRAS mutations. The majority of KRAS oncogenic mutants remain to be targeted. Extensive efforts have long been directed toward inhibiting the KRAS oncogenic protein by targeting the switch regions that interact with RAS downstream effectors. Recently, disrupting active KRAS dimers on the membrane has emerged as an alternative anticancer therapeutic strategy [23,69]. 

Nanodisc platforms have provided native lipid-bilayer membranes and revealed the structural mechanisms of action of two modulators of KRAS dimerization [14,41]: the synthetic monobody NS1 and the small compound BI2852. NS1 was shown to bind to an α–α dimerization site (α4–α5 region) of KRAS and thus inhibit the membrane-dependent formation of KRAS dimers in a competitive manner (Figure 7A). This observation is consistent with the crystal structure of the RAS-NS1 complex formed through an α–α dimer interface and with cell-based assays indicating that NS1 disrupts nanoclusters of KRAS and HRAS and inhibits RAF activation and MAPK signaling [16]. BI2852 was initially developed as a binder of KRAS–G12D [70]. The structure of the KRAS-BI2852 complex on the membrane revealed that two BI-2852 molecules bind to the β-interfaces of two RAS protomers and mediate the formation of a β-β dimer (Figure 7B), as observed in the crystal structure of membrane-free KRAS in complex with BI2852 [70]. This dimer is a signaling-incompetent conformation that blocks the effector-binding site at the β-interface of KRAS. Notably, most crystal structures of RAS in complex with inhibitors including NS1 and BI2852 have provided limited mechanistic insights into the inhibition of membrane-associated RAS because these RAS constructs lack the C-terminal membrane anchor and were crystallized in the absence of membrane mimetics. Designed ankyrin repeat proteins (DARPins), named K13 and K19, have been developed as inhibitors of KRAS-mediated cell signaling [71], although effector binding at the effector lobe of KRAS is not affected by DARPin binding to KRAS. The crystal structures of KRAS in complex with these DARPins have revealed that both K13 and K19 bind to the helix α3/loop 7/helix α4 region (α3–α4 region) at the allosteric lobe of KRAS [71], providing structural insights into the KRAS inhibition of DARPins. Notably, these interactions are incompatible with the structures of active KRAS-GTP dimers on both anionic and neutral membranes and the structure of inactive KRAS-GDP on the anionic membrane (Figure 7A), suggesting that DARPins act as competitive inhibitors of KRAS dimerization. 

The importance of KRAS membrane orientation in downstream RAF activation has been demonstrated by (1) several disease-associated mutations that alter the preferential orientation of active KRAS and impact the KRAS-RAF interaction [47] and (2) mutagenesis that stabilizes the exposed KRAS dimerization interface of the KRAS-RBD–CRD complex and thereby enhances downstream signaling [48]. Several small compounds have been reported as protein–membrane interaction (PMI) modulators to promote an orientation where the membrane occludes the effector binding site of KRAS, potentially reducing the effector activation [72,73]. The altered membrane orientation may affect the formation of KRAS dimers and larger oligomers on the membrane. Among the PMI modulators, Cmpd2 has been developed to simultaneously bind to the KRAS β-interface and the membrane, thereby stabilizing the occluded state of the effector binding site, while the KRAS dimerization site is exposed and available for binding to another KRAS molecule (Figure 7C) [72]. Structural overlays of Cmpd2-bound KRAS monomers on the membrane with three distinct KRAS dimers, as described above, exhibit positions to which the disordered C-terminal membrane anchors (K172-C185) of Cmpd2-free KRAS protomers are attached (Figure 7C). Notably, the exposure of the dimerization interface of membrane-bound KRAS monomers does not necessarily promote their dimerization. KRAS-membrane proximity may facilitate cooperativity between dimerization and C-terminal membrane anchoring of a second KRAS molecule; if a dimerization site of one KRAS protomer is far from the membrane, membrane anchoring of the opposing protomer would be unfavorable. In efforts aimed at developing bifunctional inhibitors of KRAS, the covalent inhibitor of oncogenic KRAS-G12C, called MRTX849, which stabilizes the protein in the inactive GDP-bound form, was tagged with long-chain lipids to anchor the membrane [73]. This lipid–membrane association induces an orientation in which the effector binding site of KRAS is inaccessible to downstream effectors. Additionally, the enhanced membrane association caused by the lipidation of MRTX849 restricts the lateral mobility of KRAS-G12C and impedes the formation of its dimers or nanoclusters even though the KRAS dimerization interface is exposed [73]. 

In conclusion, to develop anticancer drugs targeting membrane-associated KRAS, which has various conformations in equilibrium, the impacts that the KRAS membrane orientation can have on RAS interactions with key downstream effectors (e.g., RAFs and PI3Ks) and upstream regulators (e.g., GEFs and GAPs), as well as KRAS self-association on the membrane, should be considered. Further, KRAS binders can affect the GTPase cycling involving GTP hydrolysis and the GDP/GDP exchange of KRAS. We believe that lipid nanoparticle (e.g., nanodiscs)-based platforms will be increasingly used to screen modulators of the dimerization and PMI of KRAS and many other peripheral membrane GTPases and to provide the structural mechanism of action of modulators in native-like membrane environments. 

## Data Availability

Not applicable.
